# Soil moisture and temperature drive emergence delays associated with fire seasonality in eucalypt forests

**DOI:** 10.1093/conphys/coad093

**Published:** 2023-07-12

**Authors:** Casey Ryan, John Nikolaus Callow, Wolfgang Lewandrowski, Ryan Tangney

**Affiliations:** School of Agriculture and Environment, The University of Western Australia, 35 Stirling Highway, Crawley, WA 6009, Australia; Department of Biodiversity, Conservation and Attractions, Kings Park Science, 1 Kattidj Close, Kings Park, WA 6005, Australia; School of Biological Sciences, The University of Western Australia, 35 Stirling Highway, Crawley, WA 6009, Australia; School of Agriculture and Environment, The University of Western Australia, 35 Stirling Highway, Crawley, WA 6009, Australia; Department of Biodiversity, Conservation and Attractions, Kings Park Science, 1 Kattidj Close, Kings Park, WA 6005, Australia; School of Biological Sciences, The University of Western Australia, 35 Stirling Highway, Crawley, WA 6009, Australia; School of Agriculture and Environment, The University of Western Australia, 35 Stirling Highway, Crawley, WA 6009, Australia; Department of Biodiversity, Conservation and Attractions, Kings Park Science, 1 Kattidj Close, Kings Park, WA 6005, Australia; Centre for Ecosystem Science, School of Biological, Earth and Environmental Sciences, University of New South Wales, School of BEES, Sydney, NSW 2052, Australia

**Keywords:** Australia, ecology, fire, germination, phenology, recruitment

## Abstract

Many ecosystems are well adapted to fire, although the impacts of fire seasonality and its effect on post-fire recruitment are less well understood. Late summer or autumn fires within eucalypt forests with a Mediterranean-type climate allow for seedling emergence during the cooler and wetter seasons. The emergence and survival after spring fires may be impacted by higher soil temperatures and water stress, delaying recruitment until the subsequent winter period. During this delay, seeds may be exposed to predation and decay, which reduce the viable seed bank. This study examines post-fire recruitment dynamics in a eucalypt forest ecosystem (Northern Jarrah Forest (NJF) of southwestern Western Australia) and whether it may be vulnerable to human-induced changes to fire season. Here, we compare *in situ* post-fire seedling emergence patterns between autumn and spring burns and account for a potential ecological mechanism driving seasonal differences in emergence by determining the thermal germination requirements of seeds for 15 common species from the NJF. Our results demonstrate that 93% of species had thermal optima between 10°C and 20°C, analogous with soil temperatures measured during the germination window (late April to October). Concurrent *in situ* post-fire emergence was highest 144 days after an autumn (seasonal) fire, followed by a 10–72% decline. In contrast, there was no emergence within the first 200 days following a spring (aseasonal) fire. We conclude that aseasonal fire in the NJF can lead to a complete delay in recruitment in the first season post-fire, resulting in a lower inter-fire growth period and increasing the potential for further reductions in recruitment through seed predation and decay. The study suggests that aseasonal fire has an immediate and significant impact on initial recruitment in the NJF, but further research is required to determine any longer-term effects of this delay and its implications for fire management in southwestern Western Australia.

## Teaser

We determined the germination requirements for 15 plant species from a strongly seasonal, fire-adapted forest ecosystem and compared seedling emergence and survival after a seasonal fire and an aseasonal fire. Our results show aseasonal fire can cause a complete delay in seedling emergence due to harsh summer conditions.

## Introduction

Fire is a unique and complex disturbance that has an essential role in driving the evolution of organisms in fire-adapted ecosystems (Keeley *et al.,* 2011). It has been an important evolutionary process in ecosystems for almost as long as terrestrial vegetation has existed (Glasspool *et al.,* 2004). The human manipulation of fire has been an important cultural and practical tool for human ancestors since before the rise of *Homo sapiens* ~300 kya (Wrangham and Carmody, 2010). While fire is essential for the survival of many plant species in fire-adapted ecosystems, it is also potentially destructive for the people living in and around them. Modifying the fire regime and fire seasonality as part of fire and forest management may have detrimental ecosystem impacts (Nolan *et al.,* 2021). Modern fire managers have the challenging task of applying and managing an appropriate fire regime to protect both human life and environmental values in fire-prone forest ecosystems.

An appropriate fire regime is essential for the persistence of fire-adapted communities (Ashton and Chinner, 1999; Burrows, 2008; Nolan *et al.,* 2021). Many species in fire-adapted ecosystems rely on fire to complete their life cycle, as there are multiple benefits to timing flowering or recruitment following a fire event (Ooi, 2010; Miller *et al.,* 2019). In many fire-adapted ecosystems, prescribed fire is applied to actively manage fire regimes and fuels while balancing the goals of preserving human life and property with the conservation of biological communities (Burrows and McCaw, 2013; McCaw, 2013). To achieve these goals, a well-developed understanding of fire ecology is paramount, as vegetation responses to altered fire regimes can be temporally and spatially variable (Hobbs and Atkins, 1988; Mackenzie *et al.,* 2021). Many communities will therefore have different requirements for the frequency, intensity and seasonality of fire, which together are called the fire regime. Meeting these requirements may become more difficult to manage as rainfall decreases and mean annual temperatures increase due to anthropogenic climate change, which has extended the fire season across southwestern Western Australia (Bates *et al.,* 2008; Burrows and McCaw, 2013). This becomes increasingly challenging when meeting annual forest management spatial-area burning targets set for the purposes of fuel reduction to protect people and property (Burrows, 2008).

In fire-adapted ecosystems, seed dormancy release and subsequent germination are closely tied to fire and the post-fire environment (Ferrandis *et al.,* 1999; Tangney *et al.,* 2020*b*; Mackenzie *et al.,* 2021). Complex dormancy mechanisms ensure that once seed dormancy is overcome, germination can proceed under optimal conditions for emergence and survival (Ooi *et al.,* 2022). Germination is induced by set environmental conditions that are acutely defined by a range of suitable temperature and moisture conditions that maximize emergence success (Alvarado and Bradford, 2002) and, in some cases, further enhance germination through additional germination cues, including light- or smoke-derived chemicals (Baskin and Baskin, 2004).

**Figure 1:**
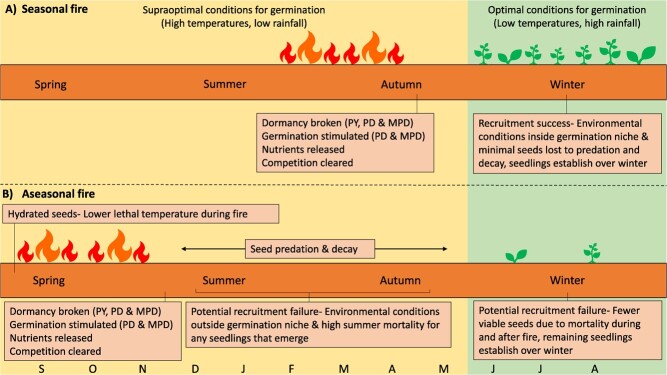
Conceptual diagram showing the expected relationship between fire and recruitment in a Mediterranean climate following (**A**) seasonal fire and (**B**) aseasonal fire. MPD = morphophysiological dormancy, PD = physiological dormancy, PY = physical dormancy (Baskin and Baskin, 2004).

The effects of fire seasonality on recruitment may be most pronounced in regions with a strongly seasonal climate like southwestern Western Australia, which has a Mediterranean climate characterized by cool, wet winters after hot, dry summers (Dell *et al.,* 1989; Miller *et al.,* 2019; Tangney *et al.,* 2022). Fire that occurs before the beginning of the cool, wet winters aligns dormancy release and germination requirements, allowing seedlings to emerge and establish over winter (Tangney *et al.,* 2020*b*), which in the Mediterranean climate of southwestern Western Australia is conducive to seedling survival; there is frequent rain, low temperatures and the flush of inorganic nutrients and reduced competition from the recent fire event (Figure 1A) (Dell *et al.,* 1989; Chambers and Attiwill, 1994). Species with physically dormant (PY) seeds are common in the Northern Jarrah Forest (NJF) and take advantage of these predictable seasons, as fire events and subsequent high soil temperatures release dormancy by breaking the hard seed coat that physically prevents germination (Baskin and Baskin, 2004). When the growing season sets in soon after a late summer or early autumn fire, seeds are non-dormant and emerge readily once germination requirements are met. For species from all dormancy classes (including non-dormancy—ND, physiological dormancy—PD and morphophysiological dormancy—MPD), it is important for successful recruitment to capitalize on these conditions through rapid seedling emergence, which maximizes growth time before the onset of summer (Ooi, 2010; Miller *et al.,* 2019).

Historical fire use by the Noongar people, the traditional owners of southwestern Western Australia, generally occurred between late spring and autumn, from November to early April (Abbott, 2002; Lullfitz *et al.,* 2017; Rodrigues *et al.,* 2022). Lightning-induced wildfires also occurred during this time (Abbott, 2002; McCaw and Read, 2012; Lullfitz *et al.,* 2017). However, due to climate change and modified land use, current prescribed burning practices are extending the fire season into the cooler and historically wetter parts of the year, particularly spring (Figure 1B) (Burrows and McCaw, 2013; Clarke *et al.,* 2013).

In this study, we assessed post-fire microsites following an autumn and (aseasonal) spring fire to evaluate the impact of fire season on post-fire germination and recruitment of a variety of common plant species in the NJF. We achieved this in two parts: first, we conducted a laboratory trial aimed at defining the thermal germination niche of common species found within the NJF. Second, a concurrent *in situ* field study tracked the emergence and survival of seedlings across two field sites, with one site tracking the emergence and survival after an autumn burn and the other tracking the emergence and survival following a spring burn. Further, we collected plot-scale temperature and moisture data at 10 plots within each site. The study asks the fundamental question: Does fire seasonality impact immediate post-fire recruitment? Within this, we consider three objectives: (i) determine the thermal germination niche for seed from 15 study species common to the NJF, (ii) compare the emergence and summer survival of seedlings in the field between autumn and spring burn sites and (iii) examine how the thermal germination niche requirements align with post-fire soil temperature and moisture dynamics.

## Materials and Methods

### Study region

The NJF is an ecological community within the Southwest Australian Floristic Region characterized by the co-dominance of two eucalypts, *Eucalyptus marginata* (Jarrah) and *Corymbia calophylla* (Marri). The community runs along the Darling Range east of Perth between the Avon River in the north and Collie in the south (Dell *et al.,* 1989). The NJF is acknowledged as under threat from drought and altered fire regimes (Lawrence *et al.,* 2022) and exists across a gradient of decreasing rainfall from 1300 mm in the southwest to 600 mm in the northeast (Dell *et al.,* 1989). The climate of the NJF is Mediterranean type, a highly seasonal rainfall regime with hot, dry summers after mild wet winters. The NJF has a varied fire history, but the current regime is dominated by low-to-moderate severity fuel-reducing prescribed burns (Dixon *et al.,* 2022) performed largely in spring, which are undertaken to maintain a mean return interval of approximately 11 years (Burrows, 2008).

### Thermal germination trial

A laboratory-based germination trial was undertaken to determine the fundamental thermal germination niche for common species in the NJF. A total of 15 common species considered likely to occur in our study region were selected for the thermal germination trial to represent a range of life forms and dormancy classes. Species with known dormancy release requirements were targeted to ensure unknown dormancy release requirements did not impact the germination trials (Table 1). Seeds of each species were collected from wild plant populations from a minimum of 10 mature plants from within the NJF and then stored at 15°C and 15% relative humidity until experimental use.

**Table 1:** Nothern Jarrah Forest species selected for the thermal germination trials

Family	Species	Dormancy type	Dormancy treatment	Seed storage
Fabaceae	*Acacia alata*	Physical	60 seconds hot water immersion	Soil
Fabaceae	*Acacia pulchella*	Physical	60 seconds hot water immersion	Soil
Haemodoraceae	*Anigozanthos manglesii*	Morphophysiological	3 hours in 100 °C oven	Soil
Fabaceae	*Bossiaea aquifolium*	Physical	45 seconds hot water immersion	Soil
Fabaceae	*Bossiaea eriocarpa*	Physical	30 seconds hot water immersion	Soil
Fabaceae	*Bossiaea ornata*	Physical	30 seconds hot water immersion	Soil
Fabaceae	*Bossiaea pulchella*	Physical	30 seconds hot water immersion	Soil
Mrytaceae	*Calothamnus sanguineus*	None	N/A	Canopy
Mrytaceae	*Corymbia calophylla*	None	N/A	Canopy
Mrytaceae	*Eucalyptus marginata*	None	N/A	Canopy
Fabaceae	*Gastrolobium capitatum*	Physical	30 seconds hot water immersion	Soil
Fabaceae	*Gompholobium tomentosum*	Physical	30 seconds hot water immersion	Soil
Fabaceae	*Hardenbergia comptoniana*	Physical	60 seconds hot water immersion	Soil
Fabaceae	*Kennedia coccinea*	Physical	30 seconds hot water immersion	Soil
Fabaceae	*Kennedia prostrata*	Physical	90 seconds hot water immersion	Soil

Seeds were X-rayed with a Faxitron MX-20 cabinet X-ray system (Hologic, Inc.) to detect whether seeds were filled, and non-filled seeds were discarded as these are non-viable. Once PY and MPD species were dormancy treated, seeds of each species (n = 500) were then surface sterilized in a 2% bleach and 1.3% tween solution to reduce fungal contamination (Elmer and Stephens, 1988). Following surface sterilization, four replicates of 25 seeds were plated on a 90 mm petri dish with 0.7% agar and placed in incubators across a range of temperatures representing expected soil temperatures in the months following fire in the NJF: 10°C, 15°C, 20°C, 25°C and 30°C (a 5°C trial was later undertaken for *Bossiaea aquifolium* and *B. pulchella* as these species appeared to have thermal germination optima of < 10°C). Seeds were incubated under a 12/12-hour light/dark regime (30μM m^−2^∙s^−1^, 400–700 nM) and scored three times a week, with germination defined as the emergence of the radicle from the seed. Scoring occurred until there was a 2-week period with no germination, at which point germination was considered complete for that species.

### Laboratory data analysis

To define the thermal germination niche, we used total germination (final germination proportion at the end of the germination trial) at each temperature in the thermal germination trial to build species-specific thermal performance curves (TPCs) using the generalized thermal performance package, *rTPC* for R (R Core Team, 2013; Padfield *et al.,* 2021). Statistical analysis was undertaken in Rstudio 2022.02.1 + 461 environment for R 4.1.2. We used each species-specific TPC to identify the modelled temperature with the highest germination (optimum germination temperature, *T*_opt_) and the lowest temperature with zero modelled germination (maximum germination temperature, *T*_max_). We also determined the range of temperatures between *T*_opt_ and *T*_max_ to indicate how buffered seeds are to increased soil temperatures following fire.

To determine the most suitable model for each species-specific response, we applied a limited suite of non-liner (nls) models defined in (Padfield *et al.,* 2021) to germination data for each species. Curve fitting suitability was assessed using Akaike information criterion (AIC, Wagenmakers and Farrell, 2004), where the lowest ranking AIC model was chosen for each species to provide best fit, unless otherwise mentioned (Supplementary Table S1). Visual checks were made for each candidate model to ensure there was minimal overfitting. Once model selection had identified the most suitable model type, models were reconstructed using the *minpack.lm* package (Elzhov *et al.,* 2022) which provides functionality for bootstrapping within the *car* package (Fox *et al.,* 2007) in R. We applied residual bootstrapping with 100 iterations for each species-specific model, which provides the ability to create species-specific 95% confidence intervals around modelled parameters as well as 95% CI around *T*_opt_ and *T*_max_.

Germination speed for each species in the thermal germination trial was modelled using non-linear functions using the ‘drm’ function available within the *drc* package (Ritz *et al.,* 2016) in R, fitting nonlinear functions as outlined in Ritz *et al.* (2016). Each model fit was tested to establish which model provided the most suitable fit, based upon log-likelihood estimations and lowest AICc ranking, and the most suitable fit was selected for each species (Supplementary Table S2-1). From these generated models we were able to estimate germination speed, defined here as time to 50% germination (T_50_) in days (Tangney *et al.,* 2020*a*).

### Field trial

**Figure 2:**
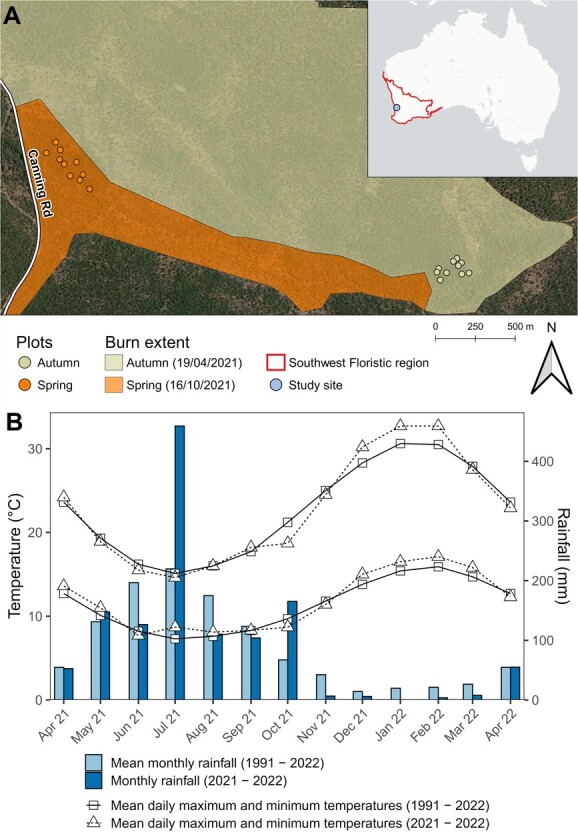
(**A**) Plot locations and burn extent for an autumn (seasonal) and spring (aseasonal) prescribed fire in Korung National Park, Western Australia. (**B**) Comparison of temperature and rainfall during the field trial with long-term temperature and rainfall at the Bickley weather station (Bureau of Meteorology, 2022).

To link laboratory models of germination dynamics with *in-situ* emergence patterns following fire we established sets of plots following two prescribed burns, the first in autumn on 19/04/2021 and another in spring on 16/10/2021, both within the NJF in southwestern Western Australia. Two sites were selected in Korung National Park, approximately 30km southeast of Perth (Figure 2a), based on their relative location to each other and the timing of applied fire. Further replication from sites across the NJF would have been preferable to ensure treatment effects were spatially reproducible, but due to time constraints and the limited number of acceptable sites with spring and autumn burns in close proximity a single autumn and spring site were selected. Average rainfall since 1991 at the nearby Bickley weather station (Lat: −32.01, Lon: 116.14) is 1146.7 mm, most of which falls in winter (Figure 2b) (Bureau of Meteorology, 2022). Both sites are characteristic of NJF communities, dominated by *E. marginata* with a typical NJF understorey largely composed of *Macrozamia riedlei, Xanthorrhoea preissii,* and many shrub species from the Fabaceae, Myrtaceae and Proteaceae families (Dell *et al.,* 1989).

Within each burn site, ten 1-m^2^ plots were installed over an area of approximately 1 ha, one month after each burn. During the first visit, all perennial woody and herbaceous seedlings were counted but could not be identified to the species level due to limited identifiable characteristics. Secondary site characteristics were visually estimated and recorded, including char height (height at which charcoal is present on tree stems), litter cover, resprouter cover and canopy cover. In subsequent months, all perennial woody and herbaceous seedlings within each plot were identified and counted monthly in the four months following the first site visit to track emergence over time. Summer survival was assessed during a final site visit in April 2022, and seedlings were again identified and counted.

To supplement the seedling emergence data and provide information on post-fire microsite suitability, a TMS-4 temperature and moisture logger (TOMST®) was installed in the centre of each plot, ensuring the loggers were in contact with the soil complex. TMS-4 loggers measure temperature at 15 cm above soil, at the soil surface, and 6 cm beneath the soil surface, as well as soil moisture at 6 cm below the soil surface, measured as volumetric soil moisture % (Wild *et al.,* 2019). As we are interested in the uppermost soil layers, where the bulk of seeds reside (Roche *et al.,* 1998), we calculated median soil temperatures, which combined the soil surface temperatures with temperatures at 6 cm into the soil every 15 minutes. We also recorded soil moisture at each plot, recorded at 6 cm into the soil. All measurements started on 08/06/2021 for the autumn burn and 24/11/2021 for the spring burn.

### Field data analysis

Following the completion of the post-fire survey, we calculated the mean, median seedling density, and species richness for each burn. Data visualization was performed with the *ggplot* (Wickham, 2011), *ggpubr* (Kassambara, 2020) and *cowplot* (Wilke, 2019) packages for R. Time series data, including logger data, species richness and seedling counts, were analysed with the R package *zoo* (Zeileis and Grothendieck, 2005) to aid visualization. Due to the complete absence of germination in any of the spring plots, formal statistical tests would have been redundant so formal statistical comparisons between spring and autumn plots were not undertaken.

## Results

### Thermal germination trial

**Figure 3:**
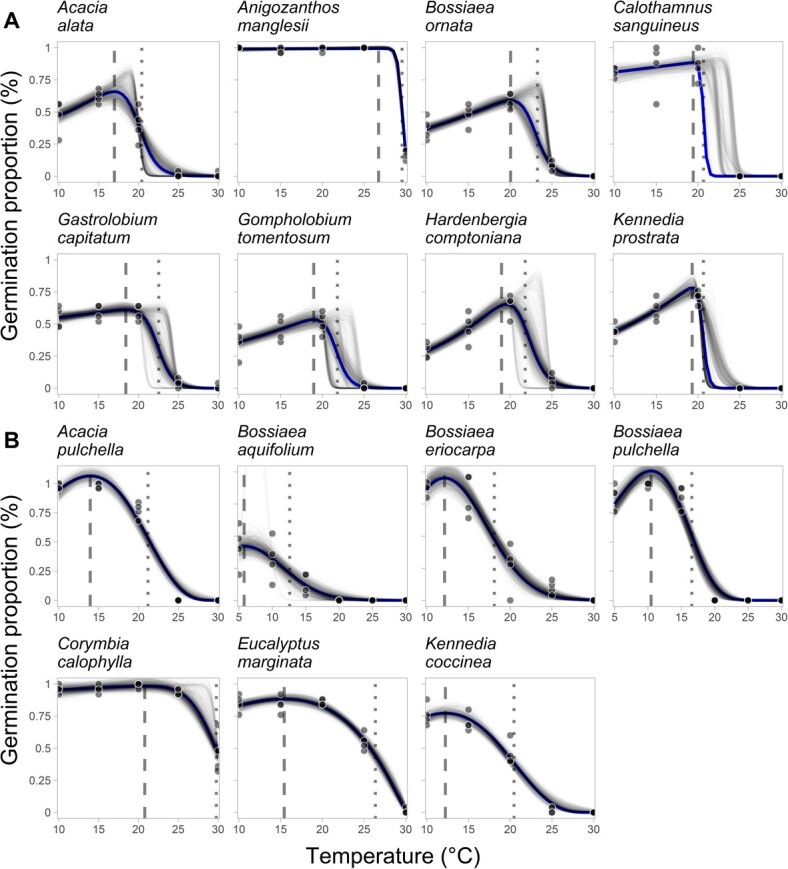
Thermal performance curves modelling the effect of incubation temperature on total germination for 15 common Northern Jarrah Forest species. The solid line represents the mean modelled estimate, partially transparent lines are bootstrapped estimates (100 iterations), dashed lines are *T*_opt_ and dotted lines are *T*_max_. (**A**) Thermal performance curves of species with narrow temperature differences between *T*_opt_ and *T*_max_. (**B**) Thermal performance curves of species with a wider temperature divide between *T*_opt_ and *T*_max_.

The 15 species showed a range of responses to the effect of incubation temperature. Two broad groups emerged (Figure 3, Supplementary Table S1). The first group is characterized by having a *T*_opt_ close the to their *T*_max_ (Figure 3A) and includes species like *Anigozanthos manglesii, Calothamnus sanguineus* and *Acacia alata*. *Bossiaea ornata* has an estimated *T*_opt_ of 20.0°C and a *T*_max_ only 3.2°C higher (Figure 3A). The other three *Bossiaea* species demonstrated some of the lowest *T*_opt_ temperatures (ranging from 5.7°C to 12.1°C), but all had a *T*_max_ > 6°C higher than their *T*_opt_ (Figure 3B). These three *Bossiaea* species are indicative of the second group of species, those with a wider temperature difference between their *T*_opt_ and their *T*_max_. This second group included both dominant tree species for the NJF, *C. calophylla* and *E. marginata* (Figure 3B).

The seeds from *A. manglesii*, *C. calophylla* and *E. marginata* all displayed high germination over a wide range of temperatures. *C. calophylla* had very high germination up to 25°C, but dropped to 46% germination at 30°C, resulting in an estimated *T*_max_ of 29.7°C (95% CI, 28.9°C–30.0°C)*,* the highest estimated *T*_max_ in this dataset, while *E. marginata* and *A. manglesii* displayed a *T*_max_ of 26.3°C and 29.5°C respectively.

All seeds germinated quickest around their *T*_opt_, but there was significant variation in germination speed across species, with Myrtaceae (ND) and Haemodoraceae (MPD) species generally germinating quicker than Fabaceae (PY) species (Supplementary Table S2-2). Nine of the 11 Fabaceae species germinated at a slow rate over a period of 40–60 days at all temperatures, resulting in longer *T*_50_ estimates than most *Myrtaceous* species. The two *Acacia* species and *Kennedia coccinea* had a fast *T*_50_ under temperatures suitable for optimum germination (*A. alata* = 9.9 days ± 0.3, *A. pulchella* = 8.3 ± 0.2, *K. coccinea* = 11.6 ± 0.5 at 15°C), but under supra-optimal germination temperatures (≥20°C) germination speed significantly declined (*A. alata* = 89.6 ± 0.3, *A. pulchella* = 24.3 ± 1.3, *K. coccinea* = 72.7 ± 6.5).

*E. marginata* took 12.4 days at 15°C to reach 50% germination, and 17.1 days at 20°C to reach 50% germination, but at 25°C germination is severely delayed, requiring 62.7 ± 2.2 days to reach 50% germination. The difference between families in near-optimum *T*_50_ was such that the Myrtaceae species with the slowest *T*_50_ near its optimum temperature (*C. sanguineus*, *T*_50_ = 12.8 ± 0.4 days at 15°C) had a similar *T*_50_ to the non-*Acacia* Fabaceae species with the fastest *T*_50_*(K. coccinea*, *T*_50_ = 11.6 ± 0.5 days at 15°C).

### Field seedling emergence and mortality

**Figure 4:**
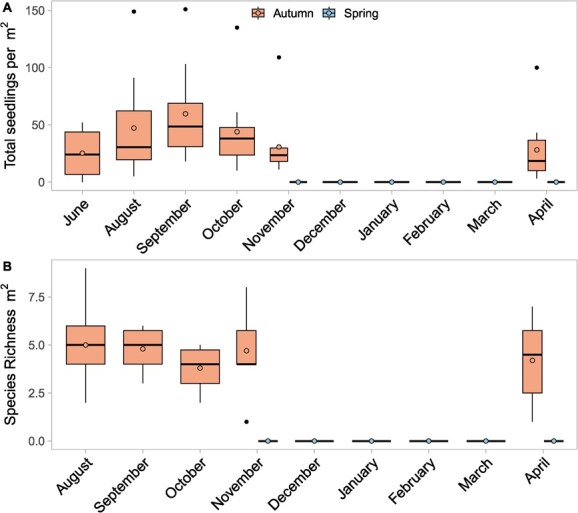
Seedling counts (**A**) and species richness (**B**) across two sites after a seasonal (autumn, 19/04/2021) and aseasonal (spring, 16/10/2021) burn in Korung National Park. Each site contained ten 1 m^2^ plots. Species richness not assessed in June after the autumn burn as seedlings could not be identified. The black horizontal lines indicate the median value, filled circles indicate mean values, the box represents the interquartile range (IQR), and the whisker extends to a maximum of 1.5 * IQR, points beyond 1.5 * IQR are visualized by black dots.

A comparison of the seedling counts and species richness between the autumn burn (19/04/2021) and the spring burn (16/10/2021) reveals a stark difference in the emergence of seedlings following fire across seasons (Figure 4). There was no emergence from any species following the spring burn (median ± SE; 0 ± 0 seedlings/m^2^), despite early rains occurring in early 2022. The final assessment for both sites occurred on 26/04/2022, by which time soil moisture at both sites had increased substantially following recent rainfall events (Figure 6). Median seedling count in the autumn site initially increased over winter to a peak of 48.5 seedlings/m^2^ 144 days after the fire, before decreasing at each following site visit to a low of 18.5 seedlings/m^2^ on the final visit 372 days after the fire. In contrast, mean seedling species richness in the autumn site peaked later at 5.8 species/m^2^ 175 days after the fire but decreased over summer to a minimum of 4.2 species/m^2^ 372 days after the fire.

**Figure 5:**
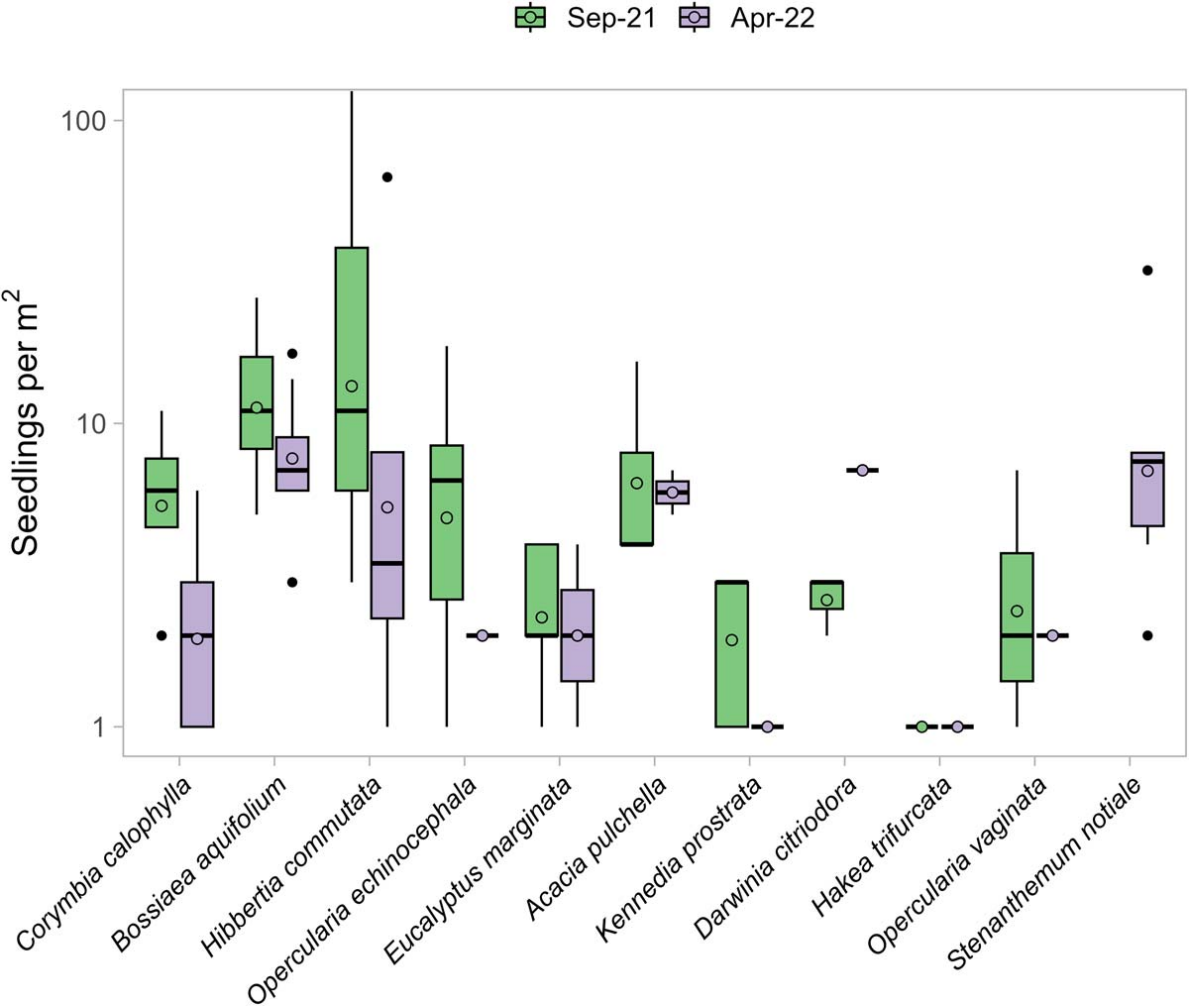
Species-level seedling counts at ten 1 m^2^ plots in Korung National Park following a seasonal (autumn) fire at peak emergence (September 2021, 144 days after fire) and after summer (April 2022, 372 days after fire). Spring burns were excluded as there were no seedlings. The black horizontal lines indicate the median value, filled circles indicate mean values, the box represents the interquartile range (IQR), and the whisker extends to a maximum of 1.5 * IQR, points beyond 1.5 * IQR are visualized by black dots.

Mean summer mortality for seedlings at the autumn site was 40.2% but varied by species (Figure 5). *Opercularia echinocephala* had the highest summer mortality, falling from a mean of 7.2 seedlings per plot at peak emergence to just 2 seedlings per plot after summer, a mortality rate of 72.1%. The species with the lowest summer mortality was *E. marginata*, which lost just 10.3% of seedlings over summer, falling from 2.6 seedlings per plot at peak emergence to 2.3 seedlings per plot after summer. *C. calophylla* (61.1% mortality), the other dominant tree species in the NJF, had much higher summer mortality than *E. marginata*, but after summer had similar mean seedlings per plot (2.4). *Darwinia citriodora* and *Stenanthemum notiale* showed higher seedling counts after summer, potentially due to delays driven by the after-ripening periods and seasonal effects found in the complex and variable dormancy responses in *Darwinia* species (Auld and Ooi, 2009). However, the germination requirements (particularly in the field) are poorly defined for both species, so the exact mechanism driving the delayed emergence is unknown. Summer mortality was not assessed at the spring site as there was no emergence.

### Soil temperature and moisture

**Figure 6:**
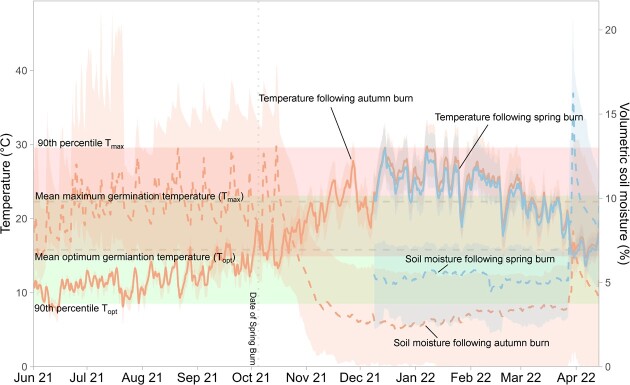
Soil temperature and soil moisture (median temperature between surface and 6 cm into the soil and moisture (measured at 6 cm below the soil surface) at two sites after seasonal (autumn) and aseasonal (spring) prescribed burns in Korung National Park. Vertical dotted line indicates date of spring burn (16/10/2021), with the autumn burn occurring before the monitoring period (19/04/2021). Temperature and moisture levels plotted using a 24-hour rolling average to remove periodicity. Shaded regions indicate 95% confidence intervals. Grey horizontal line indicates the mean *T*_max_ and *T*_opt_ for the 15 species examined while the coloured rectangles indicate 90% percentile estimates of *T*_max_ and *T*_opt_ for the 15 species examined within the laboratory trail.

Comparison of soil temperature and rainfall between the sites reveals similar soil temperature between the autumn and spring burn sites, with the autumn site logging slightly higher temperatures over summer (Figure 6). Variability in temperature between plots also increased in the autumn site over summer, before reducing again as temperatures dropped and rainfall events occurred in April 2022. Volumetric soil moisture was higher in the spring site than the autumn site, particularly after the first major rainfall event of the year in late March 2022. Soil temperature was suitable for germination between June and early December, before trending above *T*_max_ for much of the summer period. In contrast, soil moisture at both sites reduced dramatically through October and remained low until April 2022 (Figure 6).

## Discussion

Fire seasonality has a distinct and immediate impact on post-fire seedling emergence in the NJF. Seedling emergence after the autumn burn was markedly higher, when compared to spring burn plots, which experienced a complete delay in recruitment, recording zero seedlings within the first 200 days following fire. However, it is important to note that despite the immediate delay, germination and emergence following fires in spring will most likely occur during the subsequent winter (Enright and Lamont, 1989), when conditions for germination become suitable. Nevertheless, the delay in emergence exposes seeds to an array of processes before germination conditions are suitable, which in turn may reduce recruitment in subsequent seasons (Céspedes *et al.,* 2012; Ellsworth and Kauffman, 2013; Miller *et al.,* 2019, 2021; Tangney *et al.,* 2020*b*, 2022). However, the extent to which seed mortality during fire, seed predation and seed decay may have reduced the seed bank is unknown, so further research will be necessary to determine how long ungerminated seeds persist in the seed bank.

Recent research has highlighted how changes in fire season can impact post-fire recruitment (Miller *et al.,* 2019; Tangney *et al.,* 2022). Of the eight mechanisms identified in Miller *et al.* (2019) through which altered fire seasonality impacts plant survival and reproduction, the present study provides strong evidence for part of mechanism seven, post-fire seedling establishment (Miller *et al.,* 2019; Tangney *et al.,* 2020*b*). The mechanism suggests changes to fire season in strongly seasonal climates like southwestern Western Australia can reduce seedling survival by delaying germination from the early wet season to the late wet season, providing less time for growth before the onset of a dry summer. The absolute delay in seedling emergence following aseasonal fire in the present study (100%) exceeds the average of 75% in studies of post-fire seedling establishment in seasonal and weakly seasonal climates reviewed by Miller *et al.* (2019). The results here are in contrast to patterns observed in less seasonal ecosystems including temperate oceanic climates (Tangney *et al.,* 2022), which demonstrated selective delay in some species, primarily driven by seed dormancy type (Ooi, 2010). The outcome presented in this current study is consistent with results from other equally strong seasonal ecosystems (Grant, 2003; Miller *et al.,* 2021). Soil temperatures increase rapidly through November in conjunction with seasonal drought conditions, which occur as early as mid-October (Figure 6) to reduce available soil moisture and clearly delineate a defined germination window from April through to the end of October. The germination window in the NJF is substantially shortened when fires occur in spring rather than summer or autumn, potentially explaining the complete germination delay identified after the spring burn in this study.

This study raises the possibility that shifts in the predominant burn season may decrease post-fire recruitment and therefore forest resilience to climate change. Hazard reduction burning is often applied in spring for safety reasons, as spring fires are less intense and less likely to escape than fires in the historic fire season (summer and early autumn; Abbott, 2002; Burrows and McCaw, 2013). Fire seasons have also shifted in response to the decrease in rainfall, change in seasonality of rainfall and increase in temperatures experienced across southwestern Western Australia since the mid-1970s (Bates *et al.,* 2008; Clarke *et al.,* 2013). Aseasonal fire in a Mediterranean climate can cause a variety of adverse effects on recruitment, including increased seed mortality during fire and reduced seedling growth time before the onset of summer (Tangney *et al.,* 2019; Miller *et al.,* 2021). The results of this study suggest that changes to fire season have immediate adverse effects on recruitment by initiating a delay and potentially shifting community composition over time, as species from different seed dormancy classes show variable responses to the shifting regime. For example, the volumetric soil moisture was 7% to 7.4% on the day of the spring burn following a 10.6 mm rainfall event 4 days earlier, likely high enough to hydrate non-PY seeds and cause excess mortality from temperatures experienced during a cool fire (Tangney *et al.,* 2021). Such a rainfall event preceding fire would be very unlikely in a regime dominated by late summer/early autumn burns before the onset of anthropogenic climate change. Future climate change in the region is predicted to include increased temperature, decreased rainfall, increased rainfall variation (Andrys *et al.,* 2017), increased fire intensity and frequency and shifts to the fire season (Bates *et al.,* 2008; Clarke *et al.,* 2013), all of which will have impacts on recruitment in the NJF.

Two broad groups of species were identified from the thermal germination results: 1) those species whose seeds have their *T*_opt_ relatively close to their *T*_max_ (Figure 3A) and 2) those species which produce seeds with a substantially greater temperature difference between their *T*_opt_ and *T*_max_ (Figure 3B). Seeds with their *T*_opt_ close to their *T*_max_ are limiting their risk of germination at suboptimal conditions to ensure seedling survival is increased. Further, seeds with a narrow window between their optimum temperature for germination and their maximum temperature may be more exposed to recruitment failure events as soil temperatures increase in line with local ambient temperature increases as the impacts of climate change accelerate (Ooi, 2012; Ooi *et al.,* 2022). These increases in soil temperature may selectively reduce the ability for these species with narrow germination niches to germinate and emerge following fire, as post-fire soil temperatures may exceed their thermal limits. This may be further exacerbated following high severity fires (Ooi *et al.,* 2022) where large volumes of canopy biomass are lost (Dixon *et al.,* 2022), resulting in higher soil temperatures as more solar radiation hits the soil surface (Fu and Rich, 2002).

All species aside from *B. aquafolium* (*T*_opt_ = 5.7°C) had their *T*_opt_ between 10°C and 20°C, which are typical soil temperatures during winter in the NJF (Figure 6). Although most species showed a reduction in germination beyond 20°C, some species (*E. marginata, C. calophylla* and *A. manglesii*) maintained high total germination and germination rates at 25–30°C, indicating emergence after spring burns was not purely thermally limited. Median soil temperatures 6 cm below the surface never exceeded 30°C over summer, which is within the bounds of germination temperatures for some species, however emergence would be severely delayed in the upper parts of the range. However, some emergence would still be expected if emergence was primarily thermally limited, so the absence of germination following the spring burn is attributed to a combination of water stress and high soil temperatures. As temperature increases in combination with increased water stress, *C. calophylla* and *E. marginata* germination and emergence would likely be significantly impacted (McChesney *et al.,* 1995; White, 2020).

Seedling mortality following fire in autumn ranged from 10–72%, which is typical following seasonal fire (Abbott, 1984), with most seedling mortality occurring before summer. The dominant tree species in this ecosystem, namely *E. marginata* and *C. calophylla*, show divergent strategies for recruitment. *E. marginata* displayed low emergence but similarly low mortality, whereas *C. calophylla* invests more into recruitment (Abbott, 1984), which in turn increases emergence at the cost of higher seedling mortality*.* Experimental findings by Abbott (1984) also found higher emergence in *C. calophylla,* but lower summer mortality in *C. calophylla* compared to *E. marginata*. *C. calophylla* seedlings are thought to be more drought-tolerant than those of *E. marginata* (White, 2020), so the higher summer mortality for *C. calophylla* in this study may be a reflection of density-dependent competition in higher density plots where *C. calophylla* seedlings were present (Harvey *et al.,* 2011).

Soil temperatures experienced in the months following the autumn burn and the spring burn displayed very similar patterns across each site, but soil moisture was consistently higher in the spring burn site, particularly following the first major post-summer rainfall event. The spring burn site was located lower in the landscape than the autumn burn site, so landscape position may explain the differences in soil moisture (Singh *et al.,* 2021). Litter cover was reduced by a greater extent following the spring fire than following the autumn fire, and post-fire resprouter cover in the spring site was lower than in the autumn site. These physical differences between post-fire microsites may further explain the discrepancy in soil moisture, as vegetation and microbial development can be an important control on water infiltration to soils (Montaldo *et al.,* 2008).

## Conclusion

Changes in fire season as a result of climate change and prescribed burning practices have a distinct and immediate post-fire effect on plant populations in seasonal climates (Miller *et al.,* 2019; Nolan *et al.,* 2021; Tangney *et al.,* 2022). This study shows that emergence delays following aseasonal fire can be absolute in a strongly seasonal climate, pushing any germination to the following season. Laboratory trials confirm that high soil temperatures in spring are one likely cause of the immediate recruitment failure following aseasonal fire. The extent to which first-year recruitment failure will impact the future composition of the NJF is of critical importance and should be the focus of future research, so further high-resolution and longer-term studies into the impacts of aseasonal fire through time in the NJF are recommended.

## Supplementary Material

Web_Material_coad093

## Data Availability

The data underlying this article are available from the corresponding author, C.R., upon reasonable request.
