# Supplementary material for: Soil moisture and temperature drive emergence delays associated with fire seasonality in eucalypt forests
Source: Conserv Physiol. 2023 Jul 12;11(1):coad093. doi: 10.1093/conphys/coad093 (PMC10709666; doi:10.1093/conphys/coad093)
Supplement: Web_Material_coad093 [file web_material_coad093.zip › supps word.docx]

*Supplementary Table S1: Extracted values from temperature performance curves created from a thermal germination trial on 19 common species in the Northern Jarrah Forest. All species were incubated at 10, 15, 20, 25, and 30 °C, except* B. aquifolium *and* B. pulchella, *which were also incubated at 5 °C. Optimum temperature defined as the modelled temperature with the highest germination, thermal maximum defined as the lowest temperature with zero modelled germination, and thermal germination breadth defined as the range of temperatures in which a species is modelled to germinate at 50% of maximum germination or higher. Models selected from those in Padfield et al. (2021). A * indicates near maximum germination across a range of temperatures, and thus an arbitrary value for optimum temperature.*

| Species | Optimum temperature (°C) | Thermal maximum (°C) | Model |
| --- | --- | --- | --- |
| *Acacia alata* | 17.0 | 20.4 | Johnson-Lewin |
| *Acacia pulchella* | 13.9 | 21.2 | O’Neill |
| *Anigozanthos manglesii* | 26.7* | 29.5 | Johnson-Lewin |
| *Bossiaea aquifolium* | 5.7 | 12.6 | Gaussian |
| *Bossiaea eriocarpa* | 12.1 | 18.0 | Gaussian |
| *Bossiaea ornata* | 20.0 | 23.3 | Johnson-Lewin |
| *Bossiaea pulchella* | 10.4 | 16.6 | Weibull |
| *Calothamnus sanguineus* | 19.4 | 20.6 | Johnson-Lewin |
| *Corymbia calophylla* | 20.8 | 29.7 | Johnson-Lewin |
| *Eucalyptus marginata* | 15.4 | 26.4 | O’Neill |
| *Gastrolobium capitatum* | 18.4 | 22.5 | Johnson-Lewin |
| *Gompholobium tomentosum* | 18.9 | 21.8 | Johnson-Lewin |
| *Hardenbergia comptoniana* | 18.9 | 21.8 | Johnson-Lewin |
| *Kennedia coccinea* | 12.2 | 20.4 | O’Neill |
| *Kennedia prostrata* | 19.4 | 20.6 | Johnson-Lewin |

*Supplementary Table S2-1: Models selected from (Ritz et al., 2016) for thermal germination speed modelling of 15 common Northern Jarrah Forest species.*

| Species | Model (number of parameters) | | | | | |
| --- | --- | --- | --- | --- | --- | --- |
|  | 5 °C | 10 °C | 15 °C | 20 °C | 25 °C | 30 °C |
| *Acacia alata* | Not tested | Weibull (3) | Log-logistic (3) | Weibull (2) | Line | Line |
| *Acacia pulchella* | Not tested | Log-logistic (2) | Weibull (3) | Weibull (3) | NA | NA |
| *Anigozanthos manglesii* | Not tested | Weibull (2) | Weibull (3) | Weibull (2) | Weibull (2) | Weibull (3) |
| *Bossiaea aquifolium* | Weibull (2) | Weibull (2) | Weibull (2) | NA | NA | NA |
| *Bossiaea eriocarpa* | Not tested | Log-logistic (3) | Log-logistic (3) | Log-logistic (3) | Weibull (3) | NA |
| *Bossiaea ornata* | Not tested | Weibull (3) | Weibull (3) | Weibull (3) | NA | NA |
| *Bossiaea pulchella* | Log-logistic (3) | Log-logistic (2) | Weibull (3) | NA | NA | NA |
| *Calothamnus sanguineus* | Not tested | Weibull (3) | Log-logistic (3) | Weibull (3) | NA | NA |
| *Corymbia calophylla* | Not tested | Weibull (3) | Weibull (2) | Weibull (3) | Weibull (3) | Weibull (2) |
| *Eucalyptus marginata* | Not tested | Weibull (3) | Weibull (3) | Weibull (2) | Weibull (2) | Log-logistic (2) |
| *Gastrolobium capitatum* | Not tested | Weibull (3) | Log-logistic (3) | Log-logistic (3) | Weibull (3) | NA |
| *Gompholobium tomentosum* | Not tested | Weibull (3) | Weibull (3) | Log-logistic (3) | Weibull (3) | NA |
| *Hardenbergia comptoniana* | Not tested | Weibull (3) | Weibull (3) | Weibull (3) | Weibull (3) | Log-logistic (3) |
| *Kennedia coccinea* | Not tested | Weibull (3) | Weibull (3) | Weibull (2) | NA | NA |
| *Kennedia prostrata* | Not tested | Log-logistic (3) | Weibull (3) | Weibull (3) | NA | NA |

*Supplementary Table S2-2: Mean time to 50% germination for 15 common Northern Jarrah Forest species. All species incubated at a constant temperature with a 12-hr light/dark cycle.*

| Species | Time to 50% emergence (days ± standard error) | | | | | |
| --- | --- | --- | --- | --- | --- | --- |
|  | **5 °C** | **10 °C** | **15 °C** | **20 °C** | **25 °C** | **30 °C** |
| *Acacia alata* | Not tested | 12.33 ± 0.87 | 9.87 ± 0.26 | 89.58 ± 0.26 | NA | NA |
| *Acacia pulchella* | Not tested | 11.45 ± 0.44 | 8.34 ± 0.20 | 24.29 ± 1.29 | NA | NA |
| *Anigozanthos manglesii* | Not tested | 23.72 ± 0.06 | 13.82 ± 0.07 | 10.96 ± 0.12 | 14.78 ± 0.16 | 114.8 ± 112.7 |
| *Bossiaea aquifolium* | 58.58 ± 0.66 | 142.3 ± 24.59 | 117.4 ± 14.66 | NA | NA | NA |
| *Bossiaea eriocarpa* | Not tested | 26.17 ± 0.43 | 24.75 ± 1.52 | 29.14 ± 1.92 | 26.36 ± 2.08 | NA |
| *Bossiaea ornata* | Not tested | 22.10 ± 0.50 | 30.27 ± 1.52 | 36.13 ± 0.94 | NA | NA |
| *Bossiaea pulchella* | 33.26 ± 1.6 | 23.60 ± 0.39 | 23.39 ± 0.57 | NA | NA | NA |
| *Calothamnus sanguineus* | Not tested | 20.33 ± 0.11 | 12.79 ± 0.44 | 14.77 ± 0.29 | NA | NA |
| *Corymbia calophylla* | Not tested | 14.73 ± 0.47 | 10.43 ± 0.20 | 4.34 ± 0.21 | 7.00 ± 0.11 | 32.89 ± 1.52 |
| *Eucalyptus marginata* | Not tested | 14.00 ± 0.24 | 12.42 ± 0.31 | 17.81 ± 0.56 | 62.77 ± 2.25 | 772.2 ± 801.9 |
| *Gastrolobium capitatum* | Not tested | 24.61 ± 1.35 | 22.97 ± 0.93 | 18.21 ± 0.65 | 26.66 ± 3.66 | NA |
| *Gompholobium tomentosum* | Not tested | 22.91 ± 0.86 | 12.04 ± 0.83 | 14.84 ± 1.52 | 23.62 ± 5.97 | NA |
| *Hardenbergia comptoniana* | Not tested | 33.51 ± 1.22 | 25.19 ± 0.57 | 17.87 ± 0.72 | 47.58 ± 1.94 | 54.17 ± 1.82 |
| *Kennedia coccinea* | Not tested | 12.52 ± 0.58 | 11.65 ± 0.48 | 72.70 ± 6.54 | NA | NA |
| *Kennedia prostrata* | Not tested | 22.88 ± 1.30 | 18.96 ± 0.59 | 19.89 ± 0.55 | NA | NA |
