# Supplementary material for: Soil moisture and temperature drive emergence delays associated with fire seasonality in eucalypt forests
Source: Conserv Physiol. 2023 Jul 12;11(1):coad093. doi: 10.1093/conphys/coad093 (PMC10709666; doi:10.1093/conphys/coad093)
Supplement: Web_Material_coad093 [file web_material_coad093.zip › supps.pdf]

Supplementary Table S1: Extracted values from temperature performance curves created from a thermal germination trial on 19 common species in the Northern Jarrah Forest. All species were incubated at 10, 15, 20, 25, and 30 °C, except B. aquifolium *and* B. pulchella, *which were also incubated at 5 °C. Optimum temperature defined as the modelled temperature with the highest germination, thermal maximum defined as the lowest temperature with zero modelled germination, and thermal germination breadth defined as the range of temperatures in which a species is modelled to germinate at 50% of maximum germination or higher. Models selected from those in Padfield et al. (2021). A * indicates near maximum germination across a range of temperatures, and thus an arbitrary value for optimum temperature.*

**Species**	**Optimum temperature (°C)**	**Thermal maximum (°C)**	**Model**
Acacia alata	17.0	20.4	Johnson-Lewin
Acacia pulchella	13.9	21.2	O'Neill
Anigozanthos manglesii	26.7*	29.5	Johnson-Lewin
Bossiaea aquifolium	5.7	12.6	Gaussian
Bossiaea eriocarpa	12.1	18.0	Gaussian
Bossiaea ornata	20.0	23.3	Johnson-Lewin
Bossiaea pulchella	10.4	16.6	Weibull
Calothamnus sanguineus	19.4	20.6	Johnson-Lewin
Corymbia calophylla	20.8	29.7	Johnson-Lewin
Eucalyptus	15.4	26.4	O'Neill

marginata			
Gastrolobium capitatum	18.4	22.5	Johnson-Lewin
Gompholobium tomentosum	18.9	21.8	Johnson-Lewin
Hardenbergia comptoniana	18.9	21.8	Johnson-Lewin
Kennedia coccinea	12.2	20.4	O'Neill
Kennedia prostrata	19.4	20.6	Johnson-Lewin

Supplementary Table S2-1: Models selected from (Ritz et al., 2016) for thermal germination speed modelling of 15 common Northern Jarrah Forest species.

**Species**	**Model (number of parameters)**					
	5 °C	10 °C	15 °C	20 °C	25 °C	30 °C
Acacia alata	Not tested	Weibull (3)	Log-logistic (3)	Weibull (2)	Line	Line
Acacia pulchella	Not tested	Log-logistic (2)	Weibull (3)	Weibull (3)	NA	NA
Anigozanthos manglesii	Not tested	Weibull (2)	Weibull (3)	Weibull (2)	Weibull (2)	Weibull (3)
Bossiaea aquifolium	Weibull (2)	Weibull (2)	Weibull (2)	NA	NA	NA
Bossiaea eriocarpa	Not tested	Log-logistic (3)	Log-logistic (3)	Log-logistic (3)	Weibull (3)	NA
Bossiaea ornata	Not tested	Weibull (3)	Weibull (3)	Weibull (3)	NA	NA
Bossiaea pulchella	Log-logistic (3)	Log-logistic (2)	Weibull (3)	NA	NA	NA
Calothamnus sanguineus	Not tested	Weibull (3)	Log-logistic (3)	Weibull (3)	NA	NA
Corymbia calophylla	Not tested	Weibull (3)	Weibull (2)	Weibull (3)	Weibull (3)	Weibull (2)
Eucalyptus marginata	Not tested	Weibull (3)	Weibull (3)	Weibull (2)	Weibull (2)	Log-logistic (2)

Gastrolobium capitatum	Not tested	Weibull (3)	Log-logistic (3)	Log-logistic (3)	Weibull (3)	NA
Gompholobium tomentosum	Not tested	Weibull (3)	Weibull (3)	Log-logistic (3)	Weibull (3)	NA
Hardenbergia comptoniana	Not tested	Weibull (3)	Weibull (3)	Weibull (3)	Weibull (3)	Log-logistic (3)
Kennedia coccinea	Not tested	Weibull (3)	Weibull (3)	Weibull (2)	NA	NA
Kennedia prostrata	Not tested	Log-logistic (3)	Weibull (3)	Weibull (3)	NA	NA

Supplementary Table S2-2: Mean time to 50% germination for 15 common Northern Jarrah Forest species. All species incubated at a constant temperature with a 12-hr light/dark cycle.

**Species**	**Time to 50% emergence (days ± standard error)**					
	**5 °C**	**10 °C**	**15 °C**	**20 °C**	**25 °C**	**30 °C**
Acacia alata	Not tested	12.33 ± 0.87	9.87 ± 0.26	89.58 ± 0.26	NA	NA
Acacia pulchella	Not tested	11.45 ± 0.44	8.34 ± 0.20	24.29 ± 1.29	NA	NA
Anigozanthos manglesii	Not tested	23.72 ± 0.06	13.82 ± 0.07	10.96 ± 0.12	14.78 ± 0.16	114.8 ± 112.7
Bossiaea aquifolium	58.58 ± 0.66	142.3 ± 24.59	117.4 ± 14.66	NA	NA	NA
Bossiaea eriocarpa	Not tested	26.17 ± 0.43	24.75 ± 1.52	29.14 ± 1.92	26.36 ± 2.08	NA
Bossiaea ornata	Not tested	22.10 ± 0.50	30.27 ± 1.52	36.13 ± 0.94	NA	NA
Bossiaea pulchella	33.26 ± 1.6	23.60 ± 0.39	23.39 ± 0.57	NA	NA	NA
Calothamnus sanguineus	Not tested	20.33 ± 0.11	12.79 ± 0.44	14.77 ± 0.29	NA	NA

Corymbia calophylla	Not tested	14.73 ± 0.47	10.43 ± 0.20	4.34 ± 0.21	7.00 ± 0.11	32.89 ± 1.52
Eucalyptus marginata	Not tested	14.00 ± 0.24	12.42 ± 0.31	17.81 ± 0.56	62.77 ± 2.25	772.2 ± 801.9
Gastrolobium capitatum	Not tested	24.61 ± 1.35	22.97 ± 0.93	18.21 ± 0.65	26.66 ± 3.66	NA
Gompholobium tomentosum	Not tested	22.91 ± 0.86	12.04 ± 0.83	14.84 ± 1.52	23.62 ± 5.97	NA
Hardenbergia comptoniana	Not tested	33.51 ± 1.22	25.19 ± 0.57	17.87 ± 0.72	47.58 ± 1.94	54.17 ± 1.82
Kennedia coccinea	Not tested	12.52 ± 0.58	11.65 ± 0.48	72.70 ± 6.54	NA	NA
Kennedia prostrata	Not tested	22.88 ± 1.30	18.96 ± 0.59	19.89 ± 0.55	NA	NA
